# Self-Criticism: A Measure of Uncompassionate Behaviors Toward the Self, Based on the Negative Components of the Self-Compassion Scale

**DOI:** 10.3389/fpsyg.2016.01281

**Published:** 2016-08-30

**Authors:** Jesús Montero-Marín, Jorge Gaete, Marcelo Demarzo, Baltasar Rodero, Luiz C. Serrano Lopez, Javier García-Campayo

**Affiliations:** ^1^Faculty of Health and Sport Sciences, Primary Care Prevention and Health Promotion Research Network, Centro de Investigación Biomédica en Red de Salud Mental, University of Zaragoza Zaragoza, Spain; ^2^Department of Public Health and Epidemiology, Faculty of Medicine, Universidad de los Andes Santiago, Chile; ^3^Department of Population Health, London School of Hygiene and Tropical Medicine London, UK; ^4^Department of Preventive Medicine, Mente Aberta – Brazilian Center for Mindfulness and Health Promotion, Universidade Federal de São Paulo São Paulo, Brazil; ^5^Instituto Israelita de Ensino e Pesquisa do Hospital Albert Einstein São Paulo, Brazil; ^6^Clinica Rodero Santander, Spain; ^7^Miguel Servet Hospital and University of Zaragoza, Primary Care Prevention and Health Promotion Research Network, Instituto de Investigación Sanitaria Aragón (IIS Aragon), Centro de Investigación Biomédica en Red de Salud Mental, Zaragoza, Spain

**Keywords:** self-criticism, self-compassion, SCS, invariance, PCP, cross-cultural

## Abstract

**Background:** The use of the Self-Compassion Scale (SCS) as a single measure has been pointed out as problematic by many authors and its originally proposed structure has repeatedly been called into question. The negative facets of this construct are more strongly related to psychopathology than the positive indicators. The aim of this study was to evaluate and compare the different structures proposed for the SCS, including a new measure based only on the negative factors, and to assess the psychometric features of the more plausible solution.

**Method:** The study employed a cross-sectional and cross-cultural design. A sample of Brazilian (*n* = 406) and Spanish (*n* = 416) primary care professionals completed the SCS, and other questionnaires to measure psychological health-related variables. The SCS factor structure was estimated using confirmatory factor analysis by the maximum likelihood method. Internal consistency was assessed by squaring the correlation between the latent true variable and the observed variables. The relationships between the SCS and other constructs were analyzed using Spearman's *r*_*s*_.

**Results:** The structure with the best fit was comprised of the three negative first-order factors of “self-judgment”, “isolation” and “over-identification”, and one negative second-order factor, which has been named “self-criticism” [CFI = 0.92; RMSEA = 0.06 (90% CI = 0.05–0.07); SRMR = 0.05]. This solution was supported by both samples, presented partial metric invariance [CFI = 0.91; RMSEA = 0.06 (90% CI = 0.05–0.06); SRMR = 0.06], and showed significant correlations with other health-related psychological constructs. Reliability was adequate for all the dimensions (*R* ≥ 0.70).

**Conclusions:** The original structure proposed for the SCS was not supported by the data. Self-criticism, comprising only the negative SCS factors, might be a measure of uncompassionate behaviors toward the self, with good psychometric properties and practical implications from a clinical point of view, reaching a stable structure and overcoming possible methodological artifacts.

## Introduction

In recent years there has been a growing global movement that recognizes the potential role of compassion in many fields (http://charterforcompassion.org/), such as healthcare, business, education, and sports. In healthcare, several systematic reviews and meta-analyses have shown the importance of compassion in respect to psychopathology, for clinical and non-clinical populations (MacBeth and Gumley, 2012; Leaviss and Uttley, 2015; Shonin et al., 2015). Western psychological theories propose that compassion is a complex construct, which involves cognitive, affective and behavioral experiences, whose primary function is to facilitate cooperation and protection of the weak and those who suffer (Goetz et al., 2010). The Buddhist perspective understands compassion as a basic quality of human beings, rooted in the recognition of and desire to alleviate suffering, and gives rise to pro-social behaviors (Lama, 1995, 2001).

Self-compassion is one of the two subtypes of compassion, in addition to compassion for others. It has been defined as “being touched by and open to one's own suffering, not avoiding or disconnecting from it, generating the desire to alleviate one's suffering and to heal oneself with kindness” (Neff, 2003b). Three theoretical facets of self-compassion have been described (Neff, 2003a,b), represented by pairs of opposing positive and negative components, and integrated in a higher-level order under the label of their positive denomination: (+) “self-kindness” and (−) “self-judgment”; (+) “common humanity” and (−) “isolation”; (+) “mindfulness” and (−) “over-identification.” Self-kindness extends kindness to oneself, and represents an alternative to harsh judgment and self-criticism. Common humanity acknowledges one's experiences as part of the larger human experience, rather than seeing them as separating and isolating. Mindfulness represents acceptance toward uncomfortable thoughts and feelings in balanced awareness, rather than over-identifying with them. All of these components interact with each other to form the construct (Neff, 2003b). Having high levels of self-compassion is associated with several aspects of positive mental health, such as happiness, optimism, wisdom, curiosity, and emotional intelligence (Neff et al., 2007; Heffernan et al., 2010). Self-compassion also seems to be useful as a protective factor for health professionals and other workers at risk of developing burnout, by reducing perceived stress and increasing effectiveness at work (Heffernan et al., 2010; Boellinghausm et al., 2014; Raab, 2014).

Strongly based on the previous theoretical definition of self-compassion, Neff developed a 26-item scale (the Self-Compassion Scale or SCS) to measure this psychological construct, proposing a six first-order factor model with a single second-order factor of self-compassion (Neff, 2003a). Some studies have confirmed this proposal, using both clinical and non-clinical samples (Williams et al., 2014). Nevertheless, the generalizability of this structure has been called into question, and the validations of the scale in other languages have found controversial data. Some studies (including the Brazilian and Spanish validations), found the six first-order factors to have adequate psychometric properties, both in the overall sample and in sex and age subgroups. Nonetheless, the single second-order factor has not been supported (Garcia-Campayo et al., 2014; Petrocchi et al., 2014; Souza and Hutz, 2016). Other authors found that the six first-order factorial structure was not endorsed in patients with recurrent depression, adults in general, and meditators (Williams et al., 2014). Within this group of results some authors have suggested a two first-order factor solution, formed by the polarity of the positive and negative items, as two independent components named self-criticism and self-compassion (López et al., 2015). A bi-factorial model has even been proposed a posteriori as a way to justify the use of an overall self-compassion total score, arguing that self-compassion may be a mixture between the compassionate and uncompassionate ways with which individuals respond to suffering (Neff, 2016). This solution could permit to save the difficulties encountered when defending only one first-order factor of self-compassion (Neff, 2003a; Williams et al., 2014).

Noteworthy, the original theoretical framework on which the SCS was based appears to suggest a three-order structure. The first of these would be formed by the six described essential factors; the second would consist of the matching opposing components in the three previously mentioned facets; and the third would represent self-compassion as a single higher-order factor. It seems evident that the original theoretical framework would, at least, require the presence of two factorial levels, while it is not completely clear that there is need for a single score for the construct (Neff, 2003b). On the other hand, we cannot exclude the hypothesis that the weakness of the SCS in terms of factorial validity across studies might be due to methodological artifacts arising from its own structure. This structure, formed by two halves of positive and negative items, may show a certain trend to group the items according to the direction of their statements, rather than reflecting different ways to respond to suffering.

Tests comparing the strength of the relationship between the SCS and psychopathology have shown that the negative facets of the construct are more strongly linked to mental health problems than the positive indicators (Muris and Petrocchi, 2016). This does not necessarily mean that there is a problem in the definition of the construct, but it suggests that the negative facets may have a greater usefulness from a clinical perspective, and therefore it would be worthwhile to focus attention on them. A recent study (Zeng et al., 2016) showed that the original structure of the SCS was not replicated in a sample of Buddhists and of non-Buddhists; the components of self-kindness and common humanity did not show negative correlations with their opposite factors; they were not associated with better emotional outcomes; and they were not predicted by the regular practice of loving-kindness meditation. Moreover, it has been recognized that the use of the SCS total score as an individual index of self-compassion is problematic (Muris et al., 2016). All of this may decrease the relative importance of the positive facets, while pointing out that there is need for review and refinement in the assessment of the construct of self-compassion (Muris and Petrocchi, 2016; Zeng et al., 2016).

Our experience (Garcia-Campayo et al., 2014) also suggests that the negative SCS factors could play a more relevant role than the positive ones, from a psychopathological point of view. Only these negative factors might be important as true marks of vulnerability in disorders such as the burnout syndrome (Montero-Marin et al., 2016). Additionally, it has been observed that the negative factors may have different clinical correlates, and consequently, it would be worthwhile endeavoring to keep them differentiated, as different types of hostility or censure toward the self. In this sense, previous studies have pointed out that self-judgment could be related to harsh self-criticism (Zuroff et al., 1990); isolation to social withdrawal (Rubin and Coplan, 2004); and over-identification to self-focused rumination (Lyubomirsky and Nolen-Hoeksema, 1995). However, the latent structure of the negative SCS factors has never been evaluated as a possible independent solution. In the same way that the “Mindful Attention Awareness Scale” (MAAS) originally had two factors, which are not used because of their high overlapping (only the negative “lack of attention” finally remained, Brown and Ryan, 2003), we tried to explore a new approach to the assessment of the SCS, by using only the negative items. This measure might remove any possible methodological artifacts as a result of the polarized writing of the statements.

Taken independently, the negative SCS factors might constitute a brief measure of uncompassionate behaviors toward the self. This measure could be based on a three first-order factor structure, or even on a two-order structure making possible the use of an individual index of lack of self-compassion, which could be named “self-criticism.” This term may be useful when referring to all the negative components of the SCS simultaneously, as a general negative attitude toward the self. It has been previously used to refer the negative items of the SCS (López et al., 2015); it has been described as a state-trait in terms of personality, and it has been related to cognitions, affect, interpersonal goals and behavior (Zuroff et al., 2016). Nonetheless, self-criticism, would not be an alternative with the same scope as that referred to under the original term of self-compassion, given that it would not include its positive aspects as it is confined to the negative ones.

In this context, the aim of this study was to evaluate and compare the different structures proposed for the SCS so far, including new alternatives based on the positive and negative halves of the questionnaire, by assessing the psychometric features of the more plausible solution. In this respect and firstly, we tested two potential structures that could be derived deductively from the original theoretical background (Neff, 2003b):

- “*one third-order factor*” model (self-kindness, self-judgment, common humanity, isolation, mindfulness and over-identification, as first-order factors; self-kindness, common humanity, and mindfulness as second-order facets integrating the opposite factors; and self-compassion as a third-order factor).
- “*three second-order factor*” model (the six first-order factors; and self-kindness, common humanity and mindfulness as second order facets).

Secondly, we evaluated five structures proposed inductively or a posteriori, which have been assessed by the empirical research:

(c) “*one first-order factor*” model (in which all items are indicators of one overall self-compassion factor; Williams et al., 2014).
(d) “*one second-order factor*” model (the six first-order factors, and self-compassion as a second-order factor; Neff, 2003a).
(e) “*six first-order factors*” (the six first-order factors only; Garcia-Campayo et al., 2014).
(f) “*two first-order factors*” (self-compassion and self-criticism; López et al., 2015).
(g) “*bi-factor*” model (an overarching general factor in addition to the six first-order factors at the same level; Neff, 2016).

**Figure 1:**
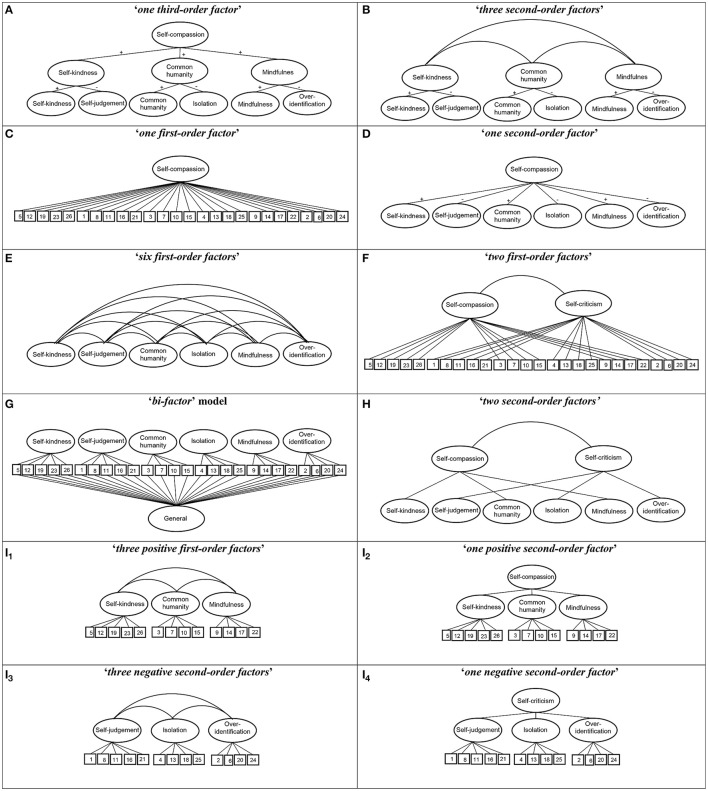
**Structures of the SCS measurement models evaluated**. The circles represent latent construct and the rectangles are observable variables. The factor loadings are represented by straight lines, and the correlations between latent factors by curved lines.

Finally, we also tested two new proposals, including some derivations according to the positive and negative halves of the questionnaire:

(h) “*two second-order factor”* model (the six first-order factors; and self-compassion and self-criticism as second-order factors), as a measure of the possible methodological artifact regarding the valence of the items, transferred to a second-order level.
(i) models formed by halves: (i_1_) “*three positive first-order factor*” model (self-kindness, common humanity and mindfulness); and (i_2_) “*one positive second-order factor*” model (three positive first-order factors, and a second-order factor of self-compassion); (i_3_) “*three negative first-order factor*” model (self-judgment, isolation and over-identification); and (i_4_) “*one negative second-order factor*” model (the three negative first-order factors, and a second-order factor of self-criticism; Figure 1).

In addition, we also aimed to evaluate possible associations with other psychological health-related variables, in order to assess the extent to which future research into the SCS may enable actions and improvements in the well-being of caregivers and the quality of primary care (PC) services. Job-related chronic distress is an occupational hazard for healthcare professionals that affects around 38% of PC personnel, and it has been linked to burnout, low health status levels, worse patient safety and poorer quality of care (Krasner et al., 2009; Al-Sareai et al., 2013; Dolan et al., 2015). There are few studies assessing potential relationships between the SCS and other important outcomes such as perceived injustice, affectivity, guilt, anxiety, depression, resilience, and awareness, despite the expected paths linking them and the need for research into the distress suffered by PC personnel (Krasner et al., 2009).

Perceived injustice is the feeling of loss, irreparability and a sense of unfairness, and it is related to poor physical health states (Rodero et al., 2012). Personal states, such as positive and negative affect, have a possible mediating role with regard to burnout (Montero-Marin et al., 2015), and guilt at work is an important correlate of this syndrome, which may worsen its symptoms (Montero-Marín et al., 2011). Anxiety and depression are important mood disorders, which were selected because of the relationships found in a previous study (Garcia-Campayo et al., 2014). Resilience is a process of adaptation to life changes that could serve as a protective factor against psychological distress and mental disorders, minimizing the consequences of occupational stress (Arrogante, 2014). Finally, awareness is the quality of paying attention to the present experience in a non-judgmental way, and is an indicator of physical and psychological health, and quality of care (Watanabe et al., 2015). It seems to be a moderator between life stressors and well-being (Atanes et al., 2015).

In short, we hypothesized at least moderate relationships between the components of the SCS and the other psychological variables, in the sense that the higher the levels of absence of self-compassion, the lower the levels of health and psychological well-being.

## Methods

### Design

An analytical cross-sectional and cross-cultural design was used for data collection in order to gain external validity, and use was made of an online platform with forced response, therefore not allowing the generation of missing data.

### Participants, procedure and ethics

The SCS was administered to two samples. The first sample was randomly recruited from the mailing list of the Aragon Health Service, in the region of Aragon, Spain, and consisted of PC professionals employed by the service between May and July 2015. The second sample was randomly recruited from the mailing list of the Brazilian Society of Family and Community Medicine, and consisted of the PC professionals who were employed during the same period in the municipalities of Santos and Santo André, Brazil. We chose to study Brazilian and Spanish samples to contrast previous validation studies in which the theoretical structure of the SCS was not fully replicated (Garcia-Campayo et al., 2014; Souza and Hutz, 2016). We selected PC personnel because of the previously described distress suffered by them (Krasner et al., 2009; Al-Sareai et al., 2013; Dolan et al., 2015), and owing to the need to develop stable constructs that facilitate the start-up and guidance of new interventions in order to reduce distress in this population. The sample size was estimated to exceed the recommended 10:1 ratio for the number of subjects to the number of test items in order to ensure its adequacy in psychometric terms (Kline, 2010). Because of the low response rate (RR) expected in this type of online designs (Kaplowitz et al., 2004), we inflated the target sample size to 1600 subjects in each group, so as to ensure that the final sample size was psychometrically adequate for the study.

A detailed e-mail message was sent to the subjects three times, at weekly intervals, explaining the objectives of the study, to whom it was directed, the voluntary nature of participation, potential benefits and risks, and data confidentiality. This message contained a link to the online survey and provided two passwords that permitted access. The protocols used were approved by the ethics committee of the regional health authorities in both countries, the Clinical Research Ethical Committee of Aragon (PI13/0084), and the Comite de Etica de la Universidade Federal de São Paulo (CAAE 30374114.1.0000.5505). The participants gave their written informed consent attesting to their willingness to participate. The study was conducted between May and July 2015. The survey data were collected anonymously.

### Measurements

#### Socio-demographics

Participants were asked about: age, sex, relationships (with or without partner), number of children, educational level (graduate, PhD), occupation (physician, nurse, other), years of service, years at last workplace, contract duration (temporary, permanent), contract type (part-time, full-time), hours worked per week, presence of economic difficulties (never, sometimes, often, almost always, always), sick leave taken in the last year (yes, no), and number of sick leave days taken in the last year (where applicable).

#### Self-compassion scale (SCS)

The SCS (Neff, 2003a) is a 26-item questionnaire designed to assess self-compassion across the subscales of self-kindness (e.g., “I try to love myself when I'm feeling emotional pain”), self-judgment (e.g., “I'm disapproving and judgmental of my flaws and inadequacies”), common humanity (e.g., “I try to see my failures as part of the human condition”), isolation (e.g., “When I'm feeling down, I tend to feel like most other people are happier than I am”), mindfulness (e.g., “when something upsets me, I try to keep my emotions in balance”) and over-identification (e.g., “when I'm feeling down, I tend to obsess and fixate on everything that is going wrong). The items assess how respondents perceive their actions toward themselves in difficult times and are rated using a Likert-type scale from 1 (almost never) to 5 (almost always). The Brazilian (Souza and Hutz, 2016) and Spanish (Garcia-Campayo et al., 2014) versions of the SCS were used.

#### Positive and negative affect schedule (PANAS)

The PANAS (Watson et al., 1988) is a self-report instrument to measure positive and negative affect. This questionnaire consists of a list of 20 adjectives, 10 per subscale (e.g., positive: “interested,” with α = 0.91; e.g., negative: “ashamed,” with α = 0.89), rated on a 5-point scale. Trait instructions (“usually”) were used in this study. This questionnaire has shown good psychometric properties in terms of reliability, factorial validity, invariance with regard to sex and age, and cross-cultural convergence (Giacomoni and Hutz, 1997; Sandín et al., 1999; López-Gómez et al., 2015), and it is one of the most widely used scales to measure mood or emotion. An “affect balance” index of “positive affect–negative affect” was also used (Diener et al., 1991).

#### Hospital anxiety and depression scale (HADS)

The HADS assesses (possible and probable) cases of anxiety and depression in a non-psychiatric population. This scale is divided into the anxiety subscale (HADS-A, with seven items, e.g., “I feel tense or wound up”; α = 0.83) and the depression subscale (HADS-D, with seven items, e.g., “I feel as if I am slowed down”; α = 0.82), both with a sensitivity and specificity of around 0.80 (Botega et al., 1995; Bjellanda et al., 2002; Castro et al., 2006).

#### Connor-davidson resilience scale (CD-RISC)

The CD-RISC (Campbell-Sills and Stein, 2007; Notario-Pacheco et al., 2011) is a 10-item measure of resilience. Each item is rated on a Likert scale from 0 (“not at all”) to 4 (“almost always”). The final score is obtained by adding the scores from the responses to each of the items (e.g., “I can deal with whatever comes my way”). Higher values indicate higher levels of resilience, with adequate internal consistency (α = 0.85), and a test-retest reliability of 0.71.

#### Injustice experiences questionnaire (IEQ)

The IEQ is a 12-item scale that asks respondents to indicate the frequency with which they have different unfairness-related thoughts (Sullivan et al., 2008). It was adapted to assess work-related perceptions of injustice, asking respondents to indicate the frequency with which they have different unfairness-related thoughts about their work (e.g., “I am suffering because of someone else's negligence”). Each question is answered using a 5-point scale from 0 (never) to 4 (all the time). On this scale, perceived injustice is assessed by only one factor, with good internal consistence (α = 0.89), and high convergence values with lack of acceptance, catastrophizing thoughts and pain (Rodero et al., 2012).

#### Mindful attention awareness scale (MAAS)

The MAAS (Brown and Ryan, 2003) is a 15-item-unidimensional measure of awareness. Each item is rated on a Likert-type scale from 1 (almost always) to 6 (almost never) in relation to the respondent's everyday experience (e.g., “I rush through activities without being really attentive to them”). Higher scores reflect higher levels of dispositional mindfulness, with appropriate internal consistence values (α = 0.89), good temporal stability and a solid unidimensional factor structure (Soler et al., 2012).

#### Visual analog scale (VAS) measuring guilt at work

We used a VAS for the purpose of measuring the level of guilt at work, a key aspect of burnout syndrome, defined as feelings of accepting the blame for one's own lack of success, desires for change and lack of responsibility (Montero-Marín et al., 2011). Participants were asked to place a mark on a point on a thermometer line that in their opinion indicated the level of guilt they were feeling. These types of visual analog scales are frequently used with adequate sensitivity/specificity, test-retest reliability, and sensitivity to change (Lesage and Berjot, 2011).

### Data analysis

Means and standard deviations, medians and interquartile ranges, frequencies and percentages were calculated to evaluate the socio-demographics, and Student *t*, Mann–Whitney U and χ^2^ tests were used to assess possible differences between samples.

Multivariate Mardia's coefficients (Mardia, 1974) and Pearson's correlation matrices (Muthén and Kaplan, 1992) were calculated to evaluate the distribution of the items. We verified the adequacy of the matrices by assessing the determinant, KMO index and Barlett's test (García et al., 2000). The fit of the models was examined using confirmatory factor analysis (CFA) by applying the maximum likelihood estimation (ML) for factor extraction (Jöreskog, 1969). We used chi-square (χ^2^), chi-square/degrees of freedom (χ^2^/*df*), the comparative fit index (CFI), the root mean square error of approximation (RMSEA) and the standardized root mean square residual (SRMR) to assess the fit of the models (Atanes et al., 2015). χ^2^ is highly sensitive to sample size (Bollen and Long, 1993), for which use was also made of χ^2^/*df*, which indicates a good fit with a value < 5 or, more strictly, < 3 (Marsh and Hocevar, 1985; Bollen and Long, 1993; Hu and Bentler, 1999; Schermelleh-Engel et al., 2003). CFI values ≥ 0.90, RMSEA ≤ 0.06, and SRMR < 0.08 indicate a good fit (Burnham and Anderson, 1998). We also calculated Akaike's criterion (AIC), as an information theory goodness-of-fit measure for the model selection. Models that generate the lowest AIC values are optimal (Burnham and Anderson, 1998).

Configurational, metric, scalar and strict invariance of the SCS model with the best fit was evaluated sequentially (Van de Schoot et al., 2012). Configurational invariance refers to the equality of the factor structure between the groups; metric invariance, to the equality of factor loadings; scalar invariance, to the equality of factor loadings and intercepts simultaneously; and strict invariance, to the equality of factor loadings, intercepts and the variance of residuals. In order to be able to accept some degree of invariance, we took into account that the restrictions on the corresponding nested models produced non-significant Δχ^2^, but mainly, owing to the sensitivity to sample size of this indicator (Hair et al., 1999), we ensured that decreases in CFI were ≤ 0.01 (Bentler, 1990; Cheung and Rensvold, 2002). Given the possible absence of invariance in the nested models, the possibility was considered of evaluating partial invariance, which would involve removing restrictions on those items with the greatest discrepancies (Vandenberg, 2002). It established that an analysis of structural equivalence would be carried out on the second-order factor weightings if the freely estimated first-order weightings did not exceed 20% (Byrne et al., 1989).

We examined the internal consistency of the factors using congeneric, tau-equivalent and parallel models of reliability (Raykov, 1997). The congeneric model assumes that each individual item measures the same latent variable, with possibly different scales, degrees of precision and magnitude of error. The tau-equivalent model implies that individual items measure the same latent variable, on the same scale, with the same degree of precision, but with possibly different degrees of error. The parallel model is the most restrictive and assumes that all items must measure the same latent variable, on the same scale, with the same degree of precision and with the same amount of error. We chose the most restrictive model with the best fit to the data (Graham, 2006). The reliability value was calculated by squaring the implied correlation between the composite latent true variable and the composite observed variable, to arrive at the percentage of the total observed variance that was accounted for by the true variable (Graham, 2006). Mean inter-item correlations and mean item-rest correlations were used, as well as the mean Spearman's *r*_*s*_ coefficients between the items over the belonging factor.

We used participants' scores in the best fitting SCS solution to evaluate the degree of association between their factors, and with regard to the other health-related psychological constructs, by means of Spearman's *r*_*s*_. The tests used were bilateral, and the significance level was α < 0.05. SPSSv19 and AMOSv20 software packages were used to perform the statistical analysis.

## Results

All materials used to produce these results are available upon request, including a detailed list of documents, data files needed, and what steps and in what sequence the interested researchers had to take in order to make this data available (King, 2013). Authors will post these materials on the group's website (Russett, 2003).

### Study participants

There were 820 participants (all were included in the analysis), of whom 406 were Brazilians, and 414 were Spanish (RR in Brazilian sample = 25.4; RR in Spanish sample = 25.9; χ^2^ = 0.12; *df* = 1; *p* = 0.731). The majority were middle-aged (mean = 45.48; SD = 11.30), women (77.8%) and university graduates (91.3%), with a partner (74.4%), and a child (median = 1; Q_1_–Q_2_ = 0–2). One-third of participants were physicians and one-third were nurses, while the remainder had other healthcare-related positions with face-to-face patient contact. The total length of service in PC was roughly two decades, with 7.93 (SD = 8.58) years at their last workplace. Some 80.2% of participants were on a permanent contract, and almost all (94.4%) worked full-time. They worked roughly 40 h/week, and almost half (42.5%) had never had economic difficulties. 26.3% had taken sick leave the previous year, with a mean of 31.45 days (SD = 60.27). Subsamples by provenance showed a large number of socio-demographic differences (Table 1).

**Table 1:** **Characteristics of study participants**.

	**Total (*n* = 820)**	**Brazilian (*n* = 406)**	**Spanish (*n* = 414)**	***p***
Age^†^	45.48 (11.30)	41.09 (10.09)	49.71 (10.78)	< 0.001
Sex^*^ (male)	185 (22.2)	63 (15.5)	122 (28.3)	< 0.001
Relationship^*^ (with partner)	623 (74.4)	286 (70.6)	337 (78.4)	0.014
Children^‡^	1 (0–2)	1 (0–2)	1 (0–2)	0.639
EDUCATION LEVEL^*^				
Graduate	765 (91.3)	374 (91.9)	391 (90.7)	0.551
PhD	72 (8.7)	33 (8.1)	39 (9.0)	
OCCUPATION^*^				
Physician	333 (39.7)	72 (17.7)	261 (60.5)	< 0.001
Nurse	228 (27.2)	62 (15.2)	166 (38.5)	
Other	277 (33.1)	273 (67.1)	4 (1.0)	
Total years of service^†^	21.02 (11.43)	17.19 (9.81)	24.66 (11.68)	< 0.001
Years at last workplace^†^	7.93 (8.58)	5.47 (5.53)	10.31 (10.21)	< 0.001
Contract duration^*^ (temporary)	166 (19.8)	39 (9.6)	127 (29.5)	< 0.001
Contract type^*^ (part-time)	47 (5.6)	41 (10.1)	6 (1.4)	< 0.001
Hours worked/week^†^	40.06 (19.71)	39.31 (26.80)	40.80 (8.19)	0.276
ECONOMIC DIFFICULTIES^*^				
Never	354 (42.5)	65 (16.0)	289 (67.8)	< 0.001
Sometimes	266 (31.9)	153 (37.6)	113 (26.5)	
Often	80 (9.6)	67 (16.5)	13 (3.1)	
Almost always	50 (6.0)	45 (11.1)	5 (1.2)	
Always	83 (10.0)	77 (18.9)	6 (1.4)	
Sick leave last year^*^ (no)	618 (73.7)	262 (64.4)	356 (82.6)	< 0.001
Number of sick leave days†	31.45 (60.27)	31.86 (64.20)	30.63 (51.99)	0.887

† means and standard deviations.

* frequencies and percentages.

‡ medians and Q_1_–Q_3_.

### Item distribution and matrices

The SCS correlation matrices for the Brazilian and Spanish samples are shown in Supplementary Material Annexes 1, 2. Mardia's index for the SCS items in the Brazilian sample was 44.93 (*p* < 0.001) [KMO = 0.92; Bartlett χ^2^ = 4940.44 (*df* = 325) *p* < 0.001; determinant < 0.001], and 30.62 (*p* < 0.001) in the Spanish sample [KMO = 0.90; Bartlett χ^2^ = 4283.59 (*df* = 325) *p* < 0.001; determinant < 0.001]. The item distribution and the correlation matrices showed adequate properties to perform the subsequent factorial analyses.

### Factorial structures

None of the models proposed for the SCS in its totality, combining positive and negative items, fully fit the data. The “*six first-order factor*” model was the one that presented the best fit, both in the Brazilian sample [χ^2^/*df* = 2.07; CFI = 0.89; RMSEA = 0.05 (90% CI = 0.04–0.06); SRMR = 0.06; AIC = 947.65], and in the Spanish sample [χ^2^/*df* = 2.05; CFI = 0.86; RMSEA = 0.05 (90% CI = 0.04–0.06); SRMR = 0.08; AIC = 993.42]. However, the fit of the “*two second-order factor*” model was very close behind, with some distance between them and the other models (Table 2).

**Table 2:** **Fit indices of the SCS models tested using CFA**.

**Models**	**χ^2^**	**df**	**χ^2/df^**	**CFI**	**RMSEA**	**LOW_90_**	**HIGH_90_**	**SRMR**	**AIC**
**A. ONE 3RD ORDER FACTOR**									
Brazilian sample	1237.71^*^	290	4.27	0.80	0.09	0.09	0.10	0.12	1359.71
Spanish sample	1261.67^*^	290	4.35	0.76	0.09	0.09	0.10	0.12	1383.66
**B. THREE 2ND ORDER FACTORS**									
Brazilian sample	1237.71^*^	290	4.27	0.80	0.09	0.09	0.10	0.12	1359.71
Spanish sample	1261.98^*^	290	4.35	0.76	0.09	0.09	0.10	0.13	1383.98
**C. ONE 1ST ORDER FACTOR**									
Brazilian sample	2266.31^*^	299	7.58	0.58	0.13	0.12	0.13	0.13	2370.31
Spanish sample	2164.46^*^	299	7.24	0.55	0.12	0.12	0.13	0.14	2268.46
**D. ONE 2ND ORDER FACTOR**									
Brazilian sample	699.29^*^	293	2.39	0.78	0.06	0.05	0.06	0.14	1439.11
Spanish sample	692.16^*^	293	2.36	0.76	0.06	0.05	0.06	0.14	1385.50
**E. SIX 1ST ORDER FACTORS**									
Brazilian sample	589.14^*^	284	2.07	0.89	0.05	0.04	0.06	0.06	947.65
Spanish sample	582.14^*^	284	2.05	0.86	0.05	0.04	0.06	0.08	993.42
**F. TWO 1ST ORDER FACTORS**									
Brazilian sample	675.07^*^	298	2.27	0.84	0.05	0.04	0.06	0.07	1179.86
Spanish sample	683.28^*^	298	2.29	0.79	0.05	0.04	0.06	0.09	1250.93
**G. BI-FACTORIAL MODEL**									
Brazilian sample	1171.22^*^	273	4.29	0.81	0.09	0.09	0.10	0.13	1327.22
Spanish sample	1056.42^*^	273	3.87	0.81	0.08	0.08	0.09	0.11	1212.42
**H. TWO 2ND ORDER FACTORS**									
Brazilian sample	858.12^*^	292	2.94	0.88	0.07	0.06	0.08	0.07	976.12
Spanish sample	949.87^*^	292	3.25	0.84	0.07	0.07	0.08	0.09	1067.87

χ^2^, minimum value of the discrepancy; df, degrees of freedom; CFI, Comparative Fit Index; RMSEA (90% CI), Root Mean Square Error of Approximation; SRMR, Standardized Root Mean Square Residual; AIC, Akaike Information Criterion. A. One 3rd-order factor model (the six 1st-order factors of self-kindness, self-judgment, common humanity, isolation, mindfulness, over-identification; the three 2nd-order factors of self-kindness, common humanity, and mindfulness as three facets integrating the opposite poles; and a self-compassion general 3rd-order factor). B. Three 2nd-order factors model (the six 1st-order factors of self-kindness, self-judgment, common humanity, isolation, mindfulness, over-identification; the three 2nd-order factors of self-kindness, common humanity and mindfulness). C. One 1st-order factor model (the one 1st-order factor of self-compassion, in which all items are indicators). D. One 2nd-order factor model (the six 1st-order factors, and self-compassion as a general 2nd-order factor). E. Six 1st-order factors model (self-kindness, self-judgment, common humanity, isolation, mindfulness, over-identification). F. Two 1st-order factors model (self-compassion and self-criticism). G. Bi-factor model (an overarching self-compassion factor, in addition to the six 1st-order factors at the same level). H. Two 2nd-order factors model (the six 1st-order factors, and self-compassion vs. self-criticism as 2nd-order factors). Graphical representations of the models are in Figure 1.

* p < 0.001.

**Figure 2:**
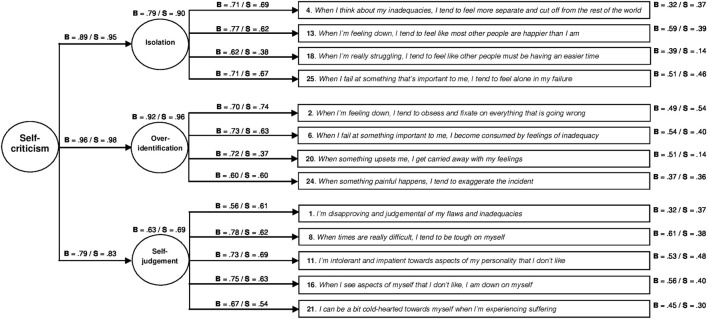
**Construct validity of the one negative second-order factor model of self-criticism**. The circles represent latent constructs and the rectangles are observable variables. The factor weightings are shown above the one-way arrows and the percentage of explained variance above the circles and boxes (standarized estimates). B, Brazilian subsample; S, Spanish subsample.

With regard to the models comprising a half of the SCS (Table 3), neither of the two models made up of the positive items was observed to adjust well, and both did so to the same degree. The negative models adjusted better that those made up of positive items, with the “*one negative second-order factor*” model (i_4_) being the one presenting the best adjustment, with good fit in all the indices, both in the Brazilian [χ^2^/*df* = 2.65; CFI = 0.93; RMSEA = 0.06 (90% CI = 0.05–0.07); SRMR = 0.05; AIC = 291.31], and in the Spanish sample [χ^2^/*df* = 2.96; CFI = 0.91; RMSEA = 0.06 (90% CI = 0.05–0.07); SRMR = 0.05; AIC = 269.95]. This configuration expained 66.8% of the variance in the Brazilian sample, and 58.5% in the Spanish sample. The CFA for the “*one negative second-order factor*” model with the unconstrained loadings and intercepts is shown in Figure 2.

**Table 3:** **Fit indices of the positive and negative halves of the SCS and invariance analysis**.

**Models**	**Δχ^2^**	**χ^2^**	**df**	**χ^2/df^**	**CFI**	**RMSEA**	**LOW_90_**	**HIGH_90_**	**SRMR**	**AIC**
**i_1_. THREE POSITIVE 1ST-ORDER FACTORS**										
Brazilians		244.04^*^	62	3.94	0.91	0.09	0.07	0.10	0.05	302.04
Spanish		285.73^*^	62	4.61	0.89	0.09	0.08	0.11	0.06	343.73
**i_2_. ONE POSITIVE 2ND-ORDER FACTOR**										
Brazilians		244.04^*^	62	3.94	0.91	0.09	0.07	0.10	0.05	302.04
Spanish		285.73^*^	62	4.61	0.89	0.09	0.08	0.11	0.06	343.73
**i_3_. THREE NEGATIVE 1ST-ORDER FACTORS**										
Brazilians		233.31^*^	62	3.76	0.93	0.08	0.07	0.09	0.06	317.31
Spanish		211.95^*^	62	3.42	0.91	0.08	0.07	0.09	0.05	295.95
**i_4_. ONE NEGATIVE 2ND-ORDER FACTOR**										
Brazilians		164.09^*^	62	2.65	0.93	0.06	0.05	0.07	0.05	291.31
Spanish		183.26^*^	62	2.96	0.91	0.06	0.05	0.07	0.05	269.95
**INVARIANCE (i_4_)**^†^										
Configurational		445.26^*^	124	3.59	0.92	0.06	0.05	0.06	0.06	613.26
Metric invariance	80.76^*^	526.02^*^	134	3.93	0.90	0.06	0.05	0.07	0.06	674.02
Scalar invariance	305.30^*^	831.32^*^	147	5.66	0.83	0.08	0.07	0.08	0.08	953.32
Full uniqueness	46.61^*^	877.93^*^	160	5.49	0.82	0.07	0.07	0.08	0.07	973.93

i_1._ Three positive first-order factors model: three positive first-order factors of self-kindness, common humanity and mindfulness. i_2_. One positive second-order factor model: the three positive first-order factors of self-kindness, common humanity and mindfulness, and self-compassion as second-order factor. i_3_. Three negative first-order factors model: three negative first-order factors of self-judgment, isolation, and over-identification. i_2_. One negative second-order factor model: the three negative first-order factors of self-judgment, isolation and over-identification, and self-criticism as second-order factor. Graphical representations of the models are in Figure 1. Δχ^2^, increase of χ^2^; χ^2^, minimum value of the discrepancy; df, degrees of freedom; CFI, Comparative Fit Index; RMSEA (90% CI), Root Mean Square Error of Approximation; SRMR, Standardized Root Mean Square Residual; AIC, Akaike Information Criterion.

* p < 0.001.

† Nested models of invariance for the one negative second-order factor model of self-criticism (i_4_).

### Invariance analysis

Table 3 shows the fit indices of the measurement invariance tests for the “*one negative second-order factor*” model (i_4_). As can be observed, the configurational model had the best trade-off between model fit and model complexity [χ^2^/*df* = 3.59; CFI = 0.92; RMSEA = 0.06 (90% CI = 0.05–0.06); SRMR = 0.06; AIC = 613.26]. The fit of metric invariance model was not bad, although Δχ^2^ gave a significant value, and the difference in CFI was excessive [Δχ^2^ = 80.76; *p* < 0.001; χ^2^/*df* = 3.93; CFI = 0.90; RMSEA = 0.06 (90% CI = 0.05–0.07); SRMR = 0.06; AIC = 674.02]. In view of this, we tried to establish partial metric invariance by assessing how loadings differed across groups. We started by releasing the load of the item with higher discrepancies (No. 20), and we found an already acceptable difference in CFI with respect to the configurational model, although Δχ^2^ continued to be significant [Δχ^2^ = 37.30; *p* < 0.001; χ^2^/*df* = 3.63; CFI = 0.91; RMSEA = 0.06 (90% CI = 0.05–0.06); SRMR = 0.06; AIC = 632.56]. When the restrictions corresponding to the second-order factor weightings were added to this model, the indices were very similar [Δχ^2^ = 46.77; *p* < 0.001; χ^2^/*df* = 3.65; CFI = 0.91; RMSEA = 0.06 (90% CI = 0.05–0.06); SRMR = 0.06; AIC = 638.03], supporting the idea of this level of invariance. When the restrictions of the intercepts were added to the weighting restrictions of the 12 selected items, the fit was unacceptable [Δχ^2^ = 279.02; *p* < 0.001; χ^2^/*df* = 5.10; CFI = 0.86; RMSEA = 0.07 (90% CI = 0.07–0.08); SRMR = 0.07; AIC = 856.28].

### Reliability

The reliability of the “*one negative second-order factor*” model (i_4_) was in an acceptable range of values in both the Brazilian and Spanish samples (Table 4). The congeneric was the reliability model with best fit to the data in all of cases. The mean inter-item correlation was 0.42 in the Brazilians and 0.34 in the Spaniards. Item-rest coefficients for the first-order factors were positive and high, with a mean of 0.61 in the Brazilians, and of 0.50 in the Spaniards. All the items were high and positively correlated to the second-order factor, with an average of 0.66 in the Brazilians, and of 0.61 in the Spaniards. The independent removal of each item was associated with lower values of reliability in all cases.

**Table 4:** **Reliability of the one negative second-order factor model of self-criticism**.

**SCS**	***R***	**χ^2^**	**df**	**χ^2/df^**	**CFI**	**RMSEA**	**LOW_90_**	**HIGH_90_**	**SRMR**	**AIC**
**SELF-CRITICISM (2ND ORDER)**										
**Brazilian sample**										
Congeneric	0.91	396.60	65	6.10	0.86	0.11	0.10	0.12	0.07	448.60
Tau-equivalent	0.91	444.52	74	6.01	0.84	0.11	0.10	0.12	0.08	478.52
Parallel	0.91	485.44	86	5.65	0.83	0.11	0.10	0.12	0.09	495.44
**Spanish sample**										
Congeneric	0.87	284.71	65	4.38	0.88	0.09	0.08	0.10	0.06	336.71
Tau-equivalent	0.87	349.55	74	4.72	0.84	0.10	0.09	0.11	0.08	383.55
Parallel	0.87	397.20	86	4.62	0.82	0.10	0.09	0.11	0.09	407.20
**SELF-JUDGMENT (1ST ORDER)**										
**Brazilian sample**										
Congeneric	0.83	10.11	5	2.02	0.99	0.05	0.01	–0.08	0.02	30.11
Tau-equivalent	0.83	41.79	9	4.64	0.95	0.10	0.07	–0.13	0.05	53.79
Parallel	0.83	52.90	13	4.07	0.94	0.09	0.06	0.11	0.07	56.90
**Spanish sample**										
Congeneric	0.76	4.26	5	0.85	1.00	0.02	0.01	0.05	0.02	24.26
Tau-equivalent	0.75	14.09	9	1.57	0.99	0.04	0.01	0.07	0.04	26.09
Parallel	0.76	21.23	13	1.63	0.98	0.04	0.01	0.07	0.03	25.22
**ISOLATION (1ST ORDER)**										
**Brazilian sample**										
Congeneric	0.80	9.14	2	4.57	0.99	0.09	0.04	0.16	0.03	25.14
Tau-equivalent	0.80	38.28	5	7.66	0.93	0.13	0.09	0.17	0.05	48.28
Parallel	0.80	43.31	8	5.41	0.93	0.10	0.08	0.14	0.06	47.31
**Spanish sample**										
Congeneric	0.71	18.99	2	9.49	0.94	0.14	0.09	0.21	0.04	34.99
Tau-equivalent	0.70	28.41	5	5.68	0.92	0.11	0.07	0.15	0.06	38.41
Parallel	0.70	33.14	8	4.14	0.91	0.08	0.06	0.12	0.06	37.14
**OVER-IDENTIFICATION (1ST ORDER)**										
**Brazilian sample**										
Congeneric	0.79	13.23	2	6.62	0.98	0.12	0.06	0.18	0.03	29.23
Tau-equivalent	0.78	26.76	5	5.35	0.95	0.10	0.07	0.14	0.05	36.76
Parallel	0.78	32.17	8	4.02	0.95	0.09	0.06	0.12	0.05	36.17
**Spanish sample**										
Congeneric	0.70	22.65	2	11.33	0.93	0.16	0.10	0.22	0.05	38.65
Tau-equivalent	0.68	55.96	5	11.19	0.82	0.16	0.12	0.20	0.08	65.96
Parallel	0.69	59.39	8	7.42	0.82	0.13	0.10	0.16	0.08	63.39

R, reliability; χ^2^, minimum value of the discrepancy; df, degrees of freedom; CFI, Comparative Fit Index; RMSEA (90% CI), Root Mean Square Error of Approximation; SRMR, Standardized Root Mean Square Residual; AIC, Akaike Information Criterion.

### Convergent/divergent validity

The associations between the negative first-order factors of self-criticism were high, with similar values in both samples: isolation–over-identification [Brazilian *r*_*s*_ = 0.73 (*p* < 0.001); Spanish *r*_*s*_ = 0.69 (*p* < 0.001)]; isolation–self-judgment [Brazilian *r*_*s*_ = 0.53 (*p* < 0.001); Spanish *r*_*s*_ = 0.54 (*p* < 0.001)]; over-identification–self-judgment [Brazilian *r*_*s*_ = 0.64 (*p* < 0.001); Spanish *r*_*s*_ = 0.64 (*p* < 0.001)]. These negative first-order factors were inversely related to positive affect, affect balance, resilience and awareness, while they were directly related to negative affect, anxiety, depression, perceived injustice, and guilt (Table 5). Self-criticism, as a negative second order factor, showed a similar pattern of relationships in both samples, with significant values in all cases.

**Table 5:** **Relationships of the one negative second-order factor model with other constructs**.

**Variables**	**Sample**	**Self-criticism (2nd order)**	**Self-judgment (1st order)**	**Isolation (1st order)**	**Over-identification (1st order)**
Positive affect	B	−0.18^***^	−0.16^***^	−0.24^***^	−0.19^***^
	S	−0.24^***^	−0.18^***^	−0.22^***^	−0.24^***^
Negative affect	B	0.51^***^	0.38^***^	0.46^***^	0.54^***^
	S	0.46^***^	0.39^***^	0.36^***^	0.44^***^
Affects balance	B	−0.45^***^	−0.29^***^	−0.44^***^	−0.48^***^
	S	−0.50^***^	−0.40^***^	−0.42^***^	−0.48^***^
Anxiety	B	0.52^***^	0.38^***^	0.46^***^	0.55^***^
Depression	B	0.46^***^	0.32^***^	0.46^***^	0.44^***^
Resilience	S	−0.39^***^	−0.21^***^	−0.40^***^	−0.41^***^
Perceived injustice	S	0.44^***^	0.31^***^	0.44^***^	0.38^***^
Awareness	S	−0.42^***^	−0.33^***^	−0.33^***^	−0.44^***^
Guilty	B	0.33^***^	0.21^***^	0.35^***^	0.32^***^

Spearman's r_s_ correlations. B, Brazilian subsample (n = 406). S, Spanish subsample (n = 414).

*** p < 0.001.

Interestingly, as we can see in Table 5, it appears that self-criticism were more related to negative affect than to positive affect. On the contrary, “self-compassion”, as a positive second order factor emerged from model i_2_, was equally related to positive and negative affect, in both Brazilian (self-compassion–positive affect, *r*_*s*_ = 0.33, *p* < 0.001; self-compassion–negative affect, *r*_*s*_ = −0.34, *p* < 0.001), and Spanish (self-compassion–positive affect, *r*_*s*_ = 0.23, *p* < 0.001; self-compassion–negative affect, *r*_*s*_ = −0.27, *p* < 0.001).

## Discussion

To our knowledge, this is the first study to assess 12 possible factor structures of the SCS in a cross-cultural design, including a new perspective that might be a measure of uncompassionate behaviors toward the self, referred to as self-criticism. This measure is based on the negative items of the questionnaire, grouped in “*one negative second-order factor*,” which does not assess self-compassion, but its opposite, as a way to overcome possible methodological difficulties, and to highlight potential vulnerability marks from a psychopathological point of view. This is of relevance because the SCS is the main comprehensive questionnaire with evidence of validation that measures self-compassion, in spite of the many questions being raised in relation to its psychometric characteristics and the way in which the SCS should be scored (Kline, 2010; Williams et al., 2014; López et al., 2015; Muris and Petrocchi, 2016).

The main strength of this study was the comprehensiveness and thoroughness of the data design and study, which allowed us to assess the cross-cultural extent and implications of the evaluated construct. It used a large sample size, recruited from two different countries such as Brazil and Spain, with diverse PC professionals, features, language and cultural background. In general terms, the results of the proposed “*one negative second-order factor*” model were replicated at the level of structure, consistency and convergence, throughout both samples, which reinforces external validity. Furthermore, data quality was controlled by eliminating possible errors in the transcription process through the use of purpose-designed software. The main limitation was that values for the considered variables were self-reported, and they may have been influenced by socially desirable responses. The degree to which this is the case, and the extent to which the negative half of the SCS can be differentiated from the positive half, is a subject that should be dealt with in future studies. On the other hand, the sample was recruited online. Despite studies that confirm the reliability of the data obtained from this source (Ritter et al., 2004), these samples might be more biased than those obtained using traditional methods. This is relevant in prevalence studies, but it seems to be less important when studying patterns of associations between variables. Moreover, the RR obtained was as high as that reached in other studies (Heiervang and Goodman, 2011).

Socio-demographic data showed important differences between Brazilian and Spanish PC samples, with a higher predominance of older, male physicians in the Spanish sample, in comparison to the Brazilian sample. Some of the differences could be attributed to the specific characteristics of the PC systems operating in the health services of both countries. Although both are universal and access-based, and use the Beveridge model of funding (Mathauer and Carrin, 2011), the PC model is more physician-based in Spain and more teamwork-based in Brazil (community-orientated PC model) (Melo et al., 2015). In addition, the PC system in Brazil was implemented more recently than in Spain, which may also explain some differences.

We have seen that the “*one second-order factor*” structure, originally proposed for the SCS (Neff, 2003a), although based on the theoretical underpinnings of self-compassion, was not supported by our data, as in the case of other studies (Garcia-Campayo et al., 2014; Petrocchi et al., 2014; López et al., 2015; Souza and Hutz, 2016). Nevertheless, the model with the worst fit was the “*one first-order factor*” model. In fact, no previous study has shown evidence of adjustment of this model (Neff, 2003a; Williams et al., 2014). Similarly, the possible theoretical derivations of the “*one third-order factor*” and the “*three second-order factors*” did not fit our Brazilian and Spanish samples, and neither did its recent adaptation of the “*bi-factor*” model (Neff, 2016). On the contrary, the model that showed the best fit to the complete SCS was the “*six first-order factor*” model, as in the case of the original Spanish and Brazilian validations (Garcia-Campayo et al., 2014; Souza and Hutz, 2016). This model was followed closely by the “*two second-order factor*” model, and behind it, without too wide a gap, appeared the “*two first-order factor*” model, followed at quite a great distance by the other models, which paradoxically were closer to the original theoretical framework (Neff, 2003b).

These results cast doubt on the original design of the scale, at least in non-clinical populations and non-Anglophone contexts. That design suggests the differentiation of six first-order factors, integrated in a single, high-level dimension, directly for positive factors, and inversely for negative factors (Neff, 2003a). Subsequently, it was considered that these six first-order factors could work on the same level together with a general dimension that was able to reflect all of their characteristics (Neff, 2016). However, according to our results, the differentiation between the six first-order factors may mean that they have no possibility of integration. They may even be closer to being resolved by means of grouping to factors on a higher level, depending on their positive or negative valence. Something similar has already been found in other studies, although in a first-order solution, through the dichotomy of the positive items and the negative ones (López et al., 2015). This context of results could be reflecting a certain tendency to respond in a different way depending on the sense with which the items are assessed, facilitating the emergence of factors related to these response tendencies rather than describing the substantive order of the phenomenon. The same artifact has been observed in other areas of psychological research, e.g., personality (Olatunji et al., 2007; Yilmaz et al., 2008), giving rise to debates that are somewhat futile.

In an endeavor to overcome the above-mentioned digressions, there appeared the possibility of reducing the scale to one of its two halves, the positive or negative. We have already stated that from a clinical viewpoint, the negative half of the SCS may be of greater interest, given that it is strongly connected to this setting (Zuroff et al., 1990; Lyubomirsky and Nolen-Hoeksema, 1995; Rubin and Coplan, 2004; Garcia-Campayo et al., 2014; Montero-Marin et al., 2016; Muris and Petrocchi, 2016). On the other hand, our results suggest that the positive half of the scale may present a certain structural ambivalence, as it shows the same level of fit for both the one and the two order solutions. However, the “*one negative second-order factor*” (the three negative first-order factors of self-judgment, isolation and over-identification, and self-criticism as a second order factor), was the reduced SCS model with the best fit. It adjusted fairly well both for the Brazilian and the Spanish samples, with adequate structure, factor loadings, and explained variance. This negative configuration, which is not new in the general field of mindfulness (Brown and Ryan, 2003; Soler et al., 2012), supports the idea of keeping a two-level factor structure, as was originally proposed (Neff, 2003a), but it calls into question the need for maintaining a double theoretical structure based on pairs of opposite factors. In a strict sense, the “*one negative second-order factor*” structure would not be assessing self-compassion, but its opposite, self-criticism, which would collect harmful self-related behaviors. As we have seen, the selected negative SCS items measured latent variables in a reliable way, which reinforces results from previous studies (Yilmaz et al., 2008; Allen et al., 2012), although they may have been working with different scales and with different accuracy levels and error size. We have also observed that despite having good internal consistency (in other words, despite sharing a large part of the total variance), they could also be referring to other concepts simultaneously, which points to the complexity of the construct, particularly when applied to the clinical field (Muris and Petrocchi, 2016). In general, it may be a parsimonious, stable and consistent solution in psychometric terms.

The “*one negative second-order factor*” model did not show strong construct invariance between the samples. In fact, factor structure (the number of factors and the pattern of loadings) was the only similarity between them. When we assessed the measurement equivalence, we observed that the first-order factor loadings were not the same between groups, and therefore, although both samples structured the construct in the same way, they did not confer the same meaning to it. Specifically, differences between samples were found in building the over-identification component, with the Spanish sample giving less importance to getting carried away by feelings when something bothers them (item No. 20). However, a partial metric invariance across samples was found in the rest of first-order loadings, and also in the second-order weightings. Over-identification, was the component with the highest weighting over the second-order factor, and could be pointed out by exaggerating negative incidents. Isolation, turned out to be the next factor in importance, and could be reflected by feelings of loneliness in failure. Finally, there was self-judgment, and this could be noted by being intolerant with oneself in terms of personality. When the equivalence between the intercept values of the invariant items was examined, equality was not observed in the origins of the measurement scale, making it impossible to compare the mean levels of the latent variables between the groups. Other studies have compared the levels of self-compassion between different cultures and societies using the total SCS (Neff et al., 2008), but they were conducted without previously studying whether the resulting measurement scales were similar in the different contexts. Nonetheless, self-criticism could be considered an uncompassionate mental functioning toward the self, which is coherent with the absence of self-compassion (Werner et al., 2012). It would take into account only the negative SCS facets as a proxy, as a mirror image with special psychopathological vulnerability (Muris and Petrocchi, 2016), which could provide additional strength to the construct by deleting the previously mentioned possible methodological artifacts in its operationalization.

The correlations between the first-order factors of self-judgment, isolation and over-identification were very high, which support their convergent validity and the rationale of summarizing them by the second-order factor of self-criticism, according to the two-level framework (Neff, 2003a). As expected in terms of direction, this negative second-order factor was positively related to anxiety, depression, negative affect and guilt, and negatively related to awareness, resilience, positive affect and affect balance. These results are in line with other studies, in which self-compassion has been inversely related to stress, anxiety, depression and psychopathology (Goetz et al., 2010; Garcia-Campayo et al., 2014), and directly related to positive mental health (Neff et al., 2007; Heffernan et al., 2010). The lower magnitude found between self-criticism and positive affect, compared to negative affect, could suggest among others, the possibility of considering an independent functioning of the positive and negative halves of the SCS, all of which brings us back to the previous debate. The degree to which an inverse score for the negative half of the SCS could serve to evaluate self-compassion, aside from this debate, as occurs in other state-trait related constructs such as awareness (Brown and Ryan, 2003; Soler et al., 2012; Atanes et al., 2015), is a question that should also be resolved in future research. For this to occur, it will be necessary to gather greater evidence with which to rule out the possibility that self-compassion, beyond any artifacts, is really comprised of two independent dimensions, one positive and the other negative, as in the case of affect (Watson et al., 1988; Giacomoni and Hutz, 1997; Sandín et al., 1999; López-Gómez et al., 2015). If this were the case, the simple calculation of the balance between the positive and negative dimensions, such a subtraction from either (Diener et al., 1991), could solve the problem of a single integrated index.

Given the current views on the topic, the concept of self-criticism could be a first step to breaking the tautological cycle. Its clinical and psychopathological importance may lie in its potential ability to configure a common strategy used to deal with negative events with the idea that one's mistaken behavior is the cause of the event. This belief may give a sensation of control, and one could believe that by changing that behavior, the event will never happen again (Gilbert, 2015). However, it seems to be dysfunctional in the long-term, generating more negative affect, anxiety and depressive symptoms, and less mentally healthier states such as awareness and resilience. Both awareness and resilience have been proposed as protective factors in the development of burnout, awareness in the first phases and resilience in its advanced stages (Montero-Marin et al., 2015). A possible mechanism that could link self-criticism, as a general psychological functioning, and the development of burnout could be their associated feelings of guilt, by obstructing the protective function of awareness and resilience.

## Conclusions

The “*one negative second-order factor*” model, based on the negative half of the SCS (the first-order factors of self-judgment, isolation and over-identification, and the second-order factor of self-criticism), showed adequate psychometric properties for reliable use, at least in primary healthcare professionals in Brazil and Spain. This model was not built on a strictly comparable basis between the samples, showing possible cultural differences. The use of measures of self-criticism could show the health-related psychological functioning of PC personnel, making possible the development of future interventions to improve well-being and quality of care. However, new replication studies are needed to confirm these results and hypotheses in other countries and languages, using adequate designs to evaluate possible causal paths.

## Author contributions

JM, MD, and JGC designed the project. JG, BR, LS collected the data. JM performed the statistical analysis. All authors interpreted the results, drafted the manuscript and read and approved the final manuscript.

### Conflict of interest statement

The authors declare that the research was conducted in the absence of any commercial or financial relationships that could be construed as a potential conflict of interest.
