# Corrigendum: Self-Criticism: A Measure of Uncompassionate Behaviors Toward the Self, Based on the Negative Components of the Self-Compassion Scale

**DOI:** 10.3389/fpsyg.2020.00853

**Published:** 2020-05-05

**Authors:** Jesús Montero-Marín, Jorge Gaete, Marcelo Demarzo, Baltasar Rodero, Luiz C. Serrano Lopez, Javier García-Campayo

**Affiliations:** ^1^Faculty of Health and Sport Sciences, Primary Care Prevention and Health Promotion Research Network, Centro de Investigación Biomédica en Red de Salud Mental, University of Zaragoza, Zaragoza, Spain; ^2^Department of Public Health and Epidemiology, Faculty of Medicine, Universidad de los Andes, Santiago, Chile; ^3^Department of Population Health, London School of Hygiene and Tropical Medicine, London, United Kingdom; ^4^Department of Preventive Medicine, Mente Aberta – Brazilian Center for Mindfulness and Health Promotion, Universidade Federal de São Paulo, São Paulo, Brazil; ^5^Instituto Israelita de Ensino e Pesquisa Do Hospital Albert Einstein, São Paulo, Brazil; ^6^Clinica Rodero, Santander, Spain; ^7^Miguel Servet Hospital and University of Zaragoza, Primary Care Prevention and Health Promotion Research Network, Instituto de Investigación Sanitaria Aragón (IIS Aragon), Centro de Investigación Biomédica en Red de Salud Mental, Zaragoza, Spain

**Keywords:** self-criticism, self-compassion, SCS, invariance, PCP, cross-cultural

In the published article, there was an error in affiliation 7. Instead of “Miguel Servet Hospital and University of Zaragoza, Primary Care Prevention and Health Promotion Research Network, Instituto Aragonés de Ciencias de la Salud, Centro de Investigación Biomédica en Red de Salud Mental, Zaragoza, Spain,” it should be “Miguel Servet Hospital and University of Zaragoza, Primary Care Prevention and Health Promotion Research Network, Instituto de Investigación Sanitaria Aragón (IIS Aragon), Centro de Investigación Biomédica en Red de Salud Mental, Zaragoza, Spain.”

In addition, the **Acknowledgments** were incomplete and didn't include funding from Instituto de Salud Carlos III and the European Regional Development Fund. The fully corrected Acknowledgments appear below.

“This study has been funded by Instituto de Salud Carlos III through the project RD12/0005/0006 Co-funded by European Regional Development Fund “Una manera de hacer Europa.” JM is grateful to the Department of Preventive Medicine of the Federal University of São Paulo for the support received.”

The authors apologize for this error and state that this does not change the scientific conclusions of the article in any way. The original article has been updated.

