# Supplementary material for: Self-Criticism: A Measure of Uncompassionate Behaviors Toward the Self, Based on the Negative Components of the Self-Compassion Scale
Source: Front Psychol. 2016 Aug 30;7:1281. doi: 10.3389/fpsyg.2016.01281 (PMC5003888; doi:10.3389/fpsyg.2016.01281)
Supplement: Supplementary file 1 [file DataSheet1.DOC]

**Annex 1: SCS correlation matrix. Brazilian sample**

|  | **1** | **2** | **3** | **4** | **5** | **6** | **7** | **8** | **9** | **10** | **11** | **12** | **13** | **14** | **15** | **16** | **17** | **18** | **19** | **20** | **21** | **22** | **23** | **24** | **25** | **26** |
| --- | --- | --- | --- | --- | --- | --- | --- | --- | --- | --- | --- | --- | --- | --- | --- | --- | --- | --- | --- | --- | --- | --- | --- | --- | --- | --- |
| **SCS1** |  |  |  |  |  |  |  |  |  |  |  |  |  |  |  |  |  |  |  |  |  |  |  |  |  |  |
| **SCS2** | .498 |  |  |  |  |  |  |  |  |  |  |  |  |  |  |  |  |  |  |  |  |  |  |  |  |  |
| **SCS3** | .031 | -.077 |  |  |  |  |  |  |  |  |  |  |  |  |  |  |  |  |  |  |  |  |  |  |  |  |
| **SCS4** | .331 | .540 | -.089 |  |  |  |  |  |  |  |  |  |  |  |  |  |  |  |  |  |  |  |  |  |  |  |
| **SCS5** | -.078 | -.102 | .405 | -.128 |  |  |  |  |  |  |  |  |  |  |  |  |  |  |  |  |  |  |  |  |  |  |
| **SCS6** | .352 | .582 | -.083 | .495 | -.123 |  |  |  |  |  |  |  |  |  |  |  |  |  |  |  |  |  |  |  |  |  |
| **SCS7** | -.058 | -.068 | .417 | -.116 | .343 | -.088 |  |  |  |  |  |  |  |  |  |  |  |  |  |  |  |  |  |  |  |  |
| **SCS8** | .408 | .455 | -.102 | .480 | -.135 | .531 | -.065 |  |  |  |  |  |  |  |  |  |  |  |  |  |  |  |  |  |  |  |
| **SCS9** | .027 | -.149 | .376 | -.225 | .450 | -.151 | .315 | -.127 |  |  |  |  |  |  |  |  |  |  |  |  |  |  |  |  |  |  |
| **SCS10** | -.042 | -.038 | .373 | -.106 | .403 | -.057 | .485 | -.018 | .444 |  |  |  |  |  |  |  |  |  |  |  |  |  |  |  |  |  |
| **SCS11** | .469 | .417 | -.038 | .422 | -.121 | .451 | -.022 | .562 | -.120 | -.071 |  |  |  |  |  |  |  |  |  |  |  |  |  |  |  |  |
| **SCS12** | -.092 | -.224 | .302 | -.393 | .461 | -.288 | .254 | -.230 | .497 | .335 | -.221 |  |  |  |  |  |  |  |  |  |  |  |  |  |  |  |
| **SCS13** | .166 | .456 | -.100 | .514 | -.114 | .476 | -.083 | .381 | -.178 | -.066 | .391 | -.227 |  |  |  |  |  |  |  |  |  |  |  |  |  |  |
| **SCS14** | .019 | -.234 | .438 | -.250 | .354 | -.215 | .299 | -.164 | .503 | .300 | -.118 | .470 | -.309 |  |  |  |  |  |  |  |  |  |  |  |  |  |
| **SCS15** | -.052 | -.177 | .452 | -.308 | .406 | -.197 | .357 | -.201 | .423 | .416 | -.154 | .491 | -.218 | .653 |  |  |  |  |  |  |  |  |  |  |  |  |
| **SCS16** | .463 | .416 | -.026 | .434 | -.130 | .479 | .037 | .620 | -.117 | -.012 | .560 | -.205 | .340 | -.054 | -.150 |  |  |  |  |  |  |  |  |  |  |  |
| **SCS17** | -.061 | -.155 | .382 | -.286 | .426 | -.198 | .376 | -.199 | .473 | .514 | -.184 | .471 | -.183 | .497 | .578 | -.126 |  |  |  |  |  |  |  |  |  |  |
| **SCS18** | .107 | .354 | -.039 | .421 | -.124 | .352 | -.035 | .275 | -.190 | .007 | .257 | -.206 | .617 | -.239 | -.103 | .214 | -.065 |  |  |  |  |  |  |  |  |  |
| **SCS19** | -.128 | -.259 | .372 | -.319 | .495 | -.232 | .277 | -.300 | .358 | .328 | -.215 | .516 | -.231 | .416 | .499 | -.251 | .439 | -.127 |  |  |  |  |  |  |  |  |
| **SCS20** | .234 | .452 | -.204 | .506 | -.276 | .496 | -.154 | .426 | -.324 | -.211 | .399 | -.362 | .565 | -.381 | -.338 | .351 | -.347 | .429 | -.285 |  |  |  |  |  |  |  |
| **SCS21** | .320 | .406 | -.195 | .453 | -.196 | .444 | -.137 | .517 | -.305 | -.135 | .474 | -.383 | .436 | -.256 | -.272 | .475 | -.241 | .280 | -.330 | .558 |  |  |  |  |  |  |
| **SCS22** | -.002 | -.206 | .399 | -.242 | .444 | -.140 | .308 | -.243 | .424 | .287 | -.117 | .398 | -.172 | .446 | .417 | -.124 | .457 | -.139 | .516 | -.241 | -.229 |  |  |  |  |  |
| **SCS23** | -.266 | -.288 | .241 | -.304 | .343 | -.336 | .214 | -.322 | .384 | .253 | -.337 | .453 | -.220 | .356 | .409 | -.410 | .377 | -.189 | .447 | -.350 | -.404 | .383 |  |  |  |  |
| **SCS24** | .253 | .397 | -.120 | .423 | -.140 | .437 | -.059 | .367 | -.262 | -.129 | .359 | -.298 | .402 | -.298 | -.237 | .331 | -.219 | .349 | -.187 | .484 | .394 | -.174 | -.224 |  |  |  |
| **SCS25** | .265 | .473 | -.115 | .463 | -.113 | .496 | -.104 | .418 | -.130 | -.117 | .378 | -.250 | .552 | -.229 | -.161 | .357 | -.224 | .404 | -.202 | .534 | .454 | -.173 | -.239 | .455 |  |  |
| **SCS26** | -.053 | -.122 | .326 | -.223 | .430 | -.171 | .294 | -.177 | .402 | .346 | -.157 | .456 | -.125 | .429 | .501 | -.180 | .436 | -.112 | .478 | -.258 | -.215 | .406 | .549 | -.142 | -.074 |  |

**Annex 2: SCS correlation matrix. Spanish sample**

|  | **1** | **2** | **3** | **4** | **5** | **6** | **7** | **8** | **9** | **10** | **11** | **12** | **13** | **14** | **15** | **16** | **17** | **18** | **19** | **20** | **21** | **22** | **23** | **24** | **25** | **26** |
| --- | --- | --- | --- | --- | --- | --- | --- | --- | --- | --- | --- | --- | --- | --- | --- | --- | --- | --- | --- | --- | --- | --- | --- | --- | --- | --- |
| **SCS1** |  |  |  |  |  |  |  |  |  |  |  |  |  |  |  |  |  |  |  |  |  |  |  |  |  |  |
| **SCS2** | .491 |  |  |  |  |  |  |  |  |  |  |  |  |  |  |  |  |  |  |  |  |  |  |  |  |  |
| **SCS3** | .145 | .142 |  |  |  |  |  |  |  |  |  |  |  |  |  |  |  |  |  |  |  |  |  |  |  |  |
| **SCS4** | .339 | .589 | .134 |  |  |  |  |  |  |  |  |  |  |  |  |  |  |  |  |  |  |  |  |  |  |  |
| **SCS5** | -.079 | -.194 | .208 | -.122 |  |  |  |  |  |  |  |  |  |  |  |  |  |  |  |  |  |  |  |  |  |  |
| **SCS6** | .437 | .442 | .026 | .456 | -.089 |  |  |  |  |  |  |  |  |  |  |  |  |  |  |  |  |  |  |  |  |  |
| **SCS7** | .144 | .067 | .170 | .059 | .307 | .131 |  |  |  |  |  |  |  |  |  |  |  |  |  |  |  |  |  |  |  |  |
| **SCS8** | .367 | .393 | .063 | .348 | -.157 | .420 | .096 |  |  |  |  |  |  |  |  |  |  |  |  |  |  |  |  |  |  |  |
| **SCS9** | .012 | -.195 | .107 | -.103 | .433 | -.006 | .275 | .007 |  |  |  |  |  |  |  |  |  |  |  |  |  |  |  |  |  |  |
| **SCS10** | .093 | .079 | .103 | .068 | .194 | .033 | .448 | .017 | .329 |  |  |  |  |  |  |  |  |  |  |  |  |  |  |  |  |  |
| **SCS11** | .406 | .476 | .081 | .355 | -.080 | .437 | .090 | .413 | -.023 | .102 |  |  |  |  |  |  |  |  |  |  |  |  |  |  |  |  |
| **SCS12** | -.128 | -.234 | .200 | -.162 | .660 | -.159 | .300 | -.186 | .429 | .292 | -.175 |  |  |  |  |  |  |  |  |  |  |  |  |  |  |  |
| **SCS13** | .212 | .527 | -.030 | .430 | -.135 | .357 | .051 | .295 | -.148 | .086 | .351 | -.160 |  |  |  |  |  |  |  |  |  |  |  |  |  |  |
| **SCS14** | -.052 | -.346 | .137 | -.272 | .362 | -.136 | .157 | -.167 | .497 | .204 | -.154 | .412 | -.388 |  |  |  |  |  |  |  |  |  |  |  |  |  |
| **SCS15** | -.022 | -.210 | .178 | -.172 | .324 | -.114 | .273 | -.096 | .419 | .336 | -.146 | .388 | -.208 | .519 |  |  |  |  |  |  |  |  |  |  |  |  |
| **SCS16** | .381 | .354 | -.017 | .293 | -.107 | .472 | .016 | .395 | .012 | -.030 | .447 | -.137 | .239 | -.097 | -.054 |  |  |  |  |  |  |  |  |  |  |  |
| **SCS17** | -.094 | -.328 | .141 | -.271 | .420 | -.212 | .194 | -.177 | .493 | .185 | -.220 | .452 | -.317 | .606 | .503 | -.124 |  |  |  |  |  |  |  |  |  |  |
| **SCS18** | .146 | .225 | -.035 | .235 | .017 | .193 | .161 | .162 | .054 | .137 | .173 | -.012 | .455 | -.121 | -.011 | .159 | -.067 |  |  |  |  |  |  |  |  |  |
| **SCS19** | -.124 | -.265 | .201 | -.180 | .577 | -.091 | .286 | -.190 | .388 | .255 | -.223 | .635 | -.200 | .453 | .425 | -.167 | .507 | .044 |  |  |  |  |  |  |  |  |
| **SCS20** | .186 | .259 | .041 | .301 | .050 | .228 | .100 | .137 | -.051 | -.004 | .265 | -.043 | .204 | -.095 | -.027 | .233 | -.132 | .139 | -.054 |  |  |  |  |  |  |  |
| **SCS21** | .280 | .325 | -.031 | .307 | -.214 | .354 | -.102 | .391 | -.058 | -.051 | .371 | -.258 | .304 | -.161 | -.197 | .380 | -.181 | .194 | -.257 | .146 |  |  |  |  |  |  |
| **SCS22** | -.075 | -.214 | .164 | -.113 | .397 | -.143 | .225 | -.137 | .441 | .248 | -.177 | .458 | -.254 | .469 | .385 | -.123 | .503 | -.068 | .441 | -.027 | -.199 |  |  |  |  |  |
| **SCS23** | -.191 | -.262 | .025 | -.270 | .412 | -.230 | .162 | -.258 | .295 | .175 | -.358 | .441 | -.224 | .379 | .413 | -.312 | .468 | .027 | .449 | -.052 | -.261 | .412 |  |  |  |  |
| **SCS24** | .269 | .486 | .069 | .397 | -.161 | .283 | .064 | .334 | -.170 | -.021 | .364 | -.222 | .396 | -.299 | -.179 | .301 | -.308 | .292 | -.225 | .393 | .286 | -.199 | -.233 |  |  |  |
| **SCS25** | .347 | .481 | .142 | .422 | -.173 | .419 | .007 | .324 | -.123 | -.056 | .381 | -.210 | .366 | -.126 | -.135 | .380 | -.247 | .290 | -.134 | .299 | .332 | -.139 | -.254 | .540 |  |  |
| **SCS26** | -.217 | -.358 | .016 | -.274 | .407 | -.238 | .194 | -.258 | .364 | .233 | -.336 | .507 | -.265 | .417 | .412 | -.300 | .502 | -.073 | .446 | -.167 | -.281 | .467 | .637 | -.272 | -.244 |  |
